# Unravelling the genetic and functional diversity of dominant bacterial communities involved in manure co-composting bioremediation of complex crude oil waste sludge

**DOI:** 10.1016/j.heliyon.2022.e08945

**Published:** 2022-02-11

**Authors:** Onyedikachi Ubani, Harrison I. Atagana, Ramganesh Selvarajan, Henry JO. Ogola

**Affiliations:** a Department of Environmental Sciences, College of Agricultural and Environmental Sciences, University of South Africa, Florida Campus, Roodepoort, 1709, South Africa; b Institute of Nanotechnology & Water Sustainability, College of Science, Engineering and Technology, University of South Africa, Florida Campus, Roodepoort, 1709, South Africa; c Laboratory of Extraterrestrial Ocean Systems (LEOS), Institute of Deep-Sea Science and Engineering, Chinese Academy of Sciences, No. 28, Luhuitou Road, Sanya, 572000, Hainan Province, PR China; d School of Agricultural and Food Sciences, Jaramogi Oginga Odinga University of Science and Technology, Bondo, P.O Box 210-40601, Kenya; e PG Research Department of Microbiology, J.J College of Arts and Science (Autonomous), Sivapuram, Pudukkottai, 622 422, Tamil Nadu, India

**Keywords:** Animal manure, Bioremediation, Bacterial diversity, Co-compost, Catechol 2,3-dioxygenase

## Abstract

The present study aimed to characterize the bacterial community and functional diversity in co-composting microcosms of crude oil waste sludge amended with different animal manures, and to evaluate the scope for biostimulation based *in situ* bioremediation. Gas chromatography–mass spectrometry (GC–MS) analyses revealed enhanced attenuation (>90%) of the total polyaromatic hydrocarbons (PAHs); the manure amendments significantly enhancing (up to 30%) the degradation of high molecular weight (HMW) PAHs. Microbial community analysis showed the dominance (>99% of total sequences) of sequences affiliated to phyla *Proteobacteria*, *Firmicutes*, *Actinobacteria* and *Bacteroidetes.* The core genera enriched were related to hydrocarbon metabolism (*Pseudomonas*, *Delftia*, *Methylobacterium*, *Dietzia*, *Bacillus*, *Propionibacterium*, *Bradyrhizobium*, *Streptomyces*, *Achromobacter, Microbacterium* and *Sphingomon*as). However, manure-treated samples exhibited high number and heterogeneity of unique operational taxonomic units (OTUs) with enrichment of additional hydrocarbon-degrading bacterial taxa (*Proteiniphilum*, unclassified *Micrococcales*, unclassified *Lachnospiraceae*, *Sphingobium* and *Stenotrophomonas*). Thirty-three culturable hydrocarbon-degrading microbes were isolated from the co-composting microcosms and mainly classified into *Burkholderia, Sanguibacter, Pseudomonas, Bacillus, Rhodococcus, Lysinibacillus, Microbacterium*, *Brevibacterium*, *Geobacillus*, *Micrococcus*, *Arthrobacter*, *Cellulimicrobacterium, Streptomyces Dietzia*,etc,. that was additionally affirmed with the presence of catechol 2,3-dioxygenase gene. Finally, enhanced *in situ* degradation of total (49%), LMW (>75%) and HMW PAHs (>35%) was achieved with an enriched bacterial consortium of these microbes. Overall, these findings suggests that co-composting treatment of crude oil sludge with animal manures selects for intrinsically diverse bacterial community, that could be a driving force behind accelerated bioremediation, and can be exploited for engineered remediation processes.

## 1 Introduction

Globally, crude oil is an essential and important strategic natural energy resource for many anthropogenic activities. However, it is associated with generation of large amounts of waste from its extraction and processing such as crude oil waste sludge (COWS). Chemically, COWS is composed of low and high molecular weights polycyclic aromatic hydrocarbons (LMW/HMW-PAHs) with characteristic strong molecular bonds and hydrophobicity. These properties make them less amenable to biodegradation and are thus recalcitrant in the environment under normal conditions. Additionally, COWS and its components are substantially cytotoxic, mutagenic and carcinogenic, especially polycyclic aromatic hydrocarbons (PAHs) [1, 2]. Environmental pollution with COWS has also been linked to physical and chemical changes of natural habitats, with potential lethal and sub-lethal toxic effects on aquatics and terrestrial ecosystem [3]. As consequence, COWS has been classified as a hazardous organic contaminant that must be treated before discharge into environment [4, 5].

The challenges confronting oil refineries and petrochemical industries to meet the regulatory requirements has given rise to research interest on safe disposal and treatment technologies for crude oil wastes sludge [3]. Amongst the currently available methods, bioremediation technologies, involving the use of microorganisms to degrade crude oil waste sludge stands out as a greener approach. This is attributed to their cost-effective and less disruptive nature to the environment. These technologies focus on improving microbial growth and metabolic activity that subsequently activates the oxidation-reduction of the contaminants into simple harmless products such as water and CO_2_ [6, 7, 8, 9]. However, there is emerging paradigm that bioremediation techniques are scientifically intense procedures, owing to the inherent nutrient deficiency and recalcitrance of HMW- PAHs in crude oil sludge. This may seriously impede the catabolic activities of indigenous microorganisms and limit the rate of intrinsic bioremediation [10]. Consequently, these techniques must optimize both intrinsic and environmental conditions to promote both microbial growth and bioremediation efficiency to be tailored for specific applications and sites.

Engineered bioremediation strategies, involving amendment of oil sludge-contaminated sites with suitable nutrients (N and/or P) to improve *in-situ* microbial growth and activity to expedite PAHs bioremediation, has been demonstrated [11]. In addition, we have also previously reported that co-composting with animal manure have great potential application to decontaminate sites heavily contaminated with PAHs and crude oil [11, 12, 13]. In this technique, the introduction of organic matter improves the nutrient availability and aeration, in addition to introducing *ex situ* microbes to improve bioremediation of contaminants [14, 15, 16]. The resultant microbial activities induce elevated temperatures, which improves the solubility of contaminants and the microbial co-metabolism that degrades and transforms pollutants into humus and inert products as the compost mature. These features have made co-composting potentially popular bioremediation method, coupled with its ability to degrade of large quantities of organic pollutant at low cost with minimal environmental disruption. The potential to integrate of co-composting with other physical or chemical techniques to achieve a better and efficient biodegradation outcome has further increased interest in the technique.

Several research studies have reported the involvement of various and complex groups of *in-situ* aerobic and anaerobic bacteria and archaea for hydrocarbons biodegradation and associated nutrient recycling processes in crude oil sludge contaminated environments [10, 17, 18]. The key roles played by niche-specific guilds of known hydrocarbon utilizing aerobic/facultative anaerobic (*Mycobacterium*, *Pseudomonas*, *Longilinea*, *Geobacte*r, etc.), nitrate reducing (*Gordonia*, *Novosphigobium*, etc.) and nitrogen fixing (*Azovibrio*, *Rhodobacter*, etc.) bacteria with strictly anaerobic, fermentative, thermophilic, sulfate-reducing bacteria (*Coprothermobacter*, *Fervidobacterium, Treponema, Syntrophus, Thermodesulfovibrio, Anaerolinea Syntrophobacter, Anaerostipes Anaerobaculum*) and methanogenic archaea (such as *Methanobacterium, Methanosaeta, Thermoplasmatales,* etc.) *in situ* biodegradation technologies have been reported [17, 19]. In contrast, the *in-situ* and *ex-situ* microbial communities’ diversity, and their metabolic capability and perturbations in community composition under the co-composting environmental conditions (such as nutrient availability, temperature, pollutant surface area, oxygen content, pH, salinity, oil composition, and many more), are not yet well described. Therefore, to develop a co-composting as a tailored bioremediation technology, elucidation of a detailed composition of microbial community diversity and dynamics is essential.

In this study, a microcosm-based culture dependent and culture-independent metagenomic method was adopted to elucidate on the nature of autochthonous microbial community structure and dynamics within co-composting of COWS with different animal manures. Specifically, the effect of co-composting with pig/swine manure (SM), cow manure (CM), horse manure (HM) or poultry manure (PM) on biodegradation potential as well as change in native prokaryotic diversity composition of crude oil sludge was analysed using high throughput sequencing of 16S rRNA genes. Further, the identity and degradation potential, either individually or in consortia, of adapted endogenous bacterial populations (mesophilic, thermophilic and maturation bacteria) isolated from various manure co-compost piles with COWS was evaluated using cultural, molecular and high throughput deep sequencing of 16S rRNA genes. Thus, the study elucidated a detailed composition of bacterial community residing in co-composting pile mixtures of crude oil sludge and animal manures and explored the scope for co-composting bioremediation of COWS.

## 2 Materials and methods

### 2.1 Composting experiments

Crude oil waste sludge was collected from an oil refinery company in Durban, KwaZulu-Natal, South Africa, and its composition characterized using automated Soxhlet extraction with dichloromethane and gas chromatography/mass spectrometry (GC/MS) as described Haleyur et al. [20]. The typical PAH composition of the COWS used in this study included: 98.2 mg/kg naphthalene; 6.0 mg/kg acenaphtylene; 9.2 mg/kg acenaphthene; 27.5 mg/kg fluorene; 14.9 mg/kg phenanthrene; 41.6 mg/kg anthracene; 2.4 mg/kg fluoranthene; 14.1 mg/kg pyrene; 4.1 mg/kg benzo[a]anthracene; 54.8 mg/kg chrysene; 23.7 mg/kg benzo[b]fluoranthene; 2.6 mg/kg benzo[k]fluoranthene; 10.0 mg/kg benzo[a]pyrene; 5.1 mg/kg perylene; 10.1 mg/kg indenol(1,2,3-cd)pyrene; 11.6 mg/kg dibenzo[a,h]anthracene; 9.4 mg/kg benzo[ghi]perylene; and 3.9 mg/kg benzo[e]acephenathrylene.

Cow (CM), pig/swine (SM), horse (HM), and poultry (PM) manures were collected from the University of Pretoria farm, Onderstepoort, Pretoria, South Africa. These manures were characterized for total organic carbon (TOC), nitrogen (TN), phosphorus (TP) content using standard methods as described previously [21]. Garden soil was also collected, homogenized, air-dried and analysed to determine the soil type, TOC, TN, TP, pH and metal content [22]. Metals in the COWS and soil samples were quantified using PerkinElmer Optima 5300 DV inductively coupled plasma optical emission spectroscopy, ICP-OES (PerkinElmer Inc., Massachusetts, USA) after aqua-regia (1/3 HNO_3_–HCl, v/v) digestion as described by Sibanda et al. [23]. The average values for the physicochemical characteristics of manures and soil used for the microcosms experiments is summarized in Table 1.

**Table 1:** Characteristics of animal manures and soil used for the microcosm experiments.

Parameter	Manures				Garden soil
	PM	CM	HM	SM	
Total organic C (%)	49.2 ± 14.2	54.9 ± 5.9	52.7 ± 2.7	50.6 ± 5.9	13.01
Total N [mg/L]	277 ± 63	109 ± 8	81 ± 3	104 ± 84	3.94
Total P [mg/L]	254 ± 14	46 ± 8	50 ± 2	252 ± 29	4.4
pH					5.56
Moisture (%)					9.52
Dry matter (%)					90.48
Texture					Sandy loam
Sand (% wt)					61.3
Silt (% wt)					21.3
Clay (% wt)					9.3
Cr (mg/kg)					121.7
Pb (mg/kg)					31.9
Ni (mg/kg)					10.13
Cu (mg/kg)					38.08
Zn (mg/kg)					9.65
Mn (mg/kg)					92.38
Fe (mg/kg)					67.04
Co (mg/kg)					2.45
Mg (mg/kg)					22.37

For each co-composting treatment, 300 g COWS was initially dissolved in 400 ml of tetrachloromethane (CCl_4_, 99.55%, molar mass 153.81 g/mol, purchased from Merck Pty, South Africa), then added to 1 kg of garden soil. The resultant soil-oil sludge mixture (SSM) was mixed to a homogenous slurry, before being air-dried at room temperature to evaporate excess CCl_4_. The amended soil was mixed with wood chips in a ratio of 1:2 (w: v). For composting experiments, SSM + wood chips mixture was separately mixed with each SM, CM, HM and PM manures in a ratio of 2:1 (w:w). A portion of SSM + wood mixture with no manure supplementation was used as the control (CT). All treatments were incubated under laboratory conditions (25±5 °C, 65 ± 12% relative humidity) for a period of 10 months in triangular PVC troughs (measuring 22 cm (length) x 9.2 cm (Depth) x 20 cm (width)) with openings on the lids and sides for aeration. All treatments were replicated three times.

During the composting experiments, temperature changes and moisture content were monitored periodically. Water was added to the compost mixture when necessary to maintain moisture level between 60-80%. pH changes and carbon dioxide evolution, used to monitor microbial activities, was also measured monthly using the closed jar method as described in previous studies [24, 25]. At the end of composting, samples were collected from composts mixture for residual PAH analysis, *in-situ* PAH-degrading bacteria isolation and characterization, and metagenomic analysis. All samples for metagenomic analysis were stored at -80 °C until analysed.

### 2.2 Residual PAH analysis

Residual PAH-concentration from compost samples was recovered by extraction using automated Soxhlet technique based on EPA method 3541 [26] and dichloromethane (≥99.5%, Sigma-Aldrich) as solvent [20]. Briefly, 10 g of compost samples was transferred into a cellulose thimble and subjected to Soxhlet extraction. For each sample, a triplicate was prepared and extracted. The PAHs present in the extracts was quantified on GC/MS Agilent 7860GC system and 5975C MSD, equipped with a 7683B autosampler (Agilent Technologies Inc., California, USA). The column used was Agilent HP-5 MS ultra-inert 30 m × 0.25 mm x 0.25 μm film thickness (Agilent Technologies Inc., California, USA) and the GC-MSD conditions used for quantification were based on the optimised method described by Agilent Application Note (https://www.agilent.com/cs/library/applications/application-optimized-gc-ms-analysis-for-PAHs-in-challenging-matrices-8890-5977b-single-quadrupole-gc-ms-5994-0499en-agilent.pdf). Prior samples analysis, the GC-MSD was calibrated with 100 μg/mL PAH standards (Sigma Aldrich Ltd).

The percent PAH degradation was calculated as follows:

$$Percentage\;PAH\;degradation=\frac{\left(\left[Initial\;PAH\right]-\left[Final\;PAH\right]\right)}{\left[Intial\;PAH\right]}\times 100$$

### 2.3 Targeted 16S rDNA amplicon sequencing

#### 2.3.1 DNA extraction, library preparation and sequencing

Fifteen grams (15 g) of compost piles were suspended in 50 mL phosphate buffered saline (PBS) overnight, homogenised and centrifuged at 12,000 rpm for 5 min at 4 °C. The supernatants were subjected to total DNA extraction using the Faecal/Soil Total DNA™ extraction kit (Zymo Research Corporation, CA, USA), according to the manufacturer's protocol. The extracted DNA having A_260_:A_280_ ratios between 1.8–2.0 and concentrations of 20–150 ng/μL. The extracted DNA were amplified following a two-step PCR method; firstly using 16S rDNA 27F (5′-AGAGTTTGATCCTGGCTCAG-3′) and 1492R (5′-TACGGYTACCTTGTTACGACTT-3′) primers to cover the whole variable region; and secondly to cover the V1–V3 region using 27F and 518R primer pairs with adapter sequences that are compatible with Illumina index as described by Selvarajan et al. [27]. The resultant PCR were subsequently purified then sequenced by paired end (300 bp reads) sequencing v.3 chemistry along with its multiplex sample identifiers on the Illumina MiSeq Platform (Illumina Inc., San Diego, CA, USA) at the University of South Africa according to standard protocol.

#### 2.3.2 Bioinformatic analyses

Raw sequences were initially screened for PCR artefacts and low-quality reads using ngsShoRT (next generation sequencing Short Reads) trimmer [28], before being analyzed using Mothur v1.25 pipeline [29]. Chimeric sequences were removed using UCHIME algorithm [30]. Quality filtered non-chimeric reads were used for closed-reference picking and taxonomy assignation of Operational Taxonomic Units (OTUs) based on the SILVA SSU database release 132 (https://www.arb-silva.de/download/arb-files/), with the similarity threshold set at 0.97.

The dominant OTUs at different taxonomic levels were used to generate stacked bar charts and heatmap using *ggplot2* [31] and *heatmap.2* packages [32] in R version 3.6.1 [33], respectively, to visualize the variations and distributions of bacterial communities. Alpha diversity indices were calculated at the genetic distance of 0.03 using the plot_richness function of *phyloseq* [34]. β-diversity based Bray-Curtis dissimilarity distance and canonical correspondence analysis (CCA) to visualize the community relationships between and within each composting treatment with explanatory environmental variables was also performed using *vegan* package [35].

### 2.4 Culture dependent microbiological analyses

#### 2.4.1 Isolation of crude oil degrading bacteria

Samples consisting of 15 g of compost piles with COWS and different manures and control were suspended in 100 ml sterile mineral salt media (MSM) supplemented with 10 ml of crude oil sludge as sole source of carbon in 250 mL conical flask. The MSM stock contained in 1 L solution: 500 mg KH_2_PO_4_, 500 mg MgSO_4_. 7H_2_O, 500 mg NaH_2_PO_4_.H_2_O, 500 mg NH_4_Cl, 4000 mg NaCl, 500 mg NaHCO_3,_ 500 mg Na_2_CO_3_ and 1 mL trace element mix. Trace element mix contained in mg L^−1^: 1500 mg FeCl_2_.H_2_O, 9000 mg NaCl, 197 mg MnCl_2_. 4H_2_O, 900 mg CaCl_2_, 238 mg CoCl_2_.H_2_O, 17 mg CuCl_2_.H_2_O, 287 mg ZnSO_4_, 50 mg AlCl_3_, 62 mg H_3_BO_3_, 24 mg NiCl_2_.6H_2_O, filter-sterilised through 0.2 μm Millipore filter membrane. The flasks were incubated in the dark at 28 °C on a rotary shaker at 150 rpm for 21 days. At the end incubation, 1 mL aliquots of the enrichment cultures were aseptically transferred into new 250 mL flasks containing 100 ml sterile MSM spiked with 10 mL crude oil sludge, and again incubated for another 21 days at 28 °C on a rotary shaker in the dark. Crude oil-degrading bacteria were isolated from the enrichment cultures by serial dilutions (10^−3^-10^−8^) and spread on mineral salts agar (MSA) plates supplemented with 1% crude oil sludge. The plates were incubated for 21–28 days at 28 °C in the dark.

Distinct colonies were purified by streaking several times on nutrient agar plates to obtain pure single colonies. The pure colonies were again screened for their ability to grow and utilise crude oil by streaking on MSA plates overlaid with 1.5% oil sludge, and the plates incubated at 37 °C for 3–7 days. All the positive isolates were sub-cultured on nutrient broth at 28 °C for three days and the culture used for DNA extraction for identification, catechol 2,3-dioxygenase gene screening, PAH biodegradation screening with 2,6-dichlorophenol indophenol (2,6-DCPIP) test and bacterial consortia development for COWS degradation.

#### 2.4.2 PAH biodegradation screening test

Cell growth and PAH degradation ability of isolates that exhibited hydrocarbonoclastic activity (utilising oil sludge as sole carbon source) were further checked by rapid colorimetric test based on 2,6-dichlorophenol indophenol (2,6-DCPIP) reduction [36]. Each isolate was cultured in Bushnell Hass (BH) broth for 24 h at 37 °C while being shaken at 180 rpm. After 24 h, the culture was supplemented with a sterile mixture of 0.5% (w/v) 2,6-dichlorophenol indophenol (2,6-DCPIP), 0.1% Tween 80 and 3% (v/v) of crude oil sludge and further subcultured for 7 days at 28 °C. The degradation of crude oil sludge was monitored daily by colour change from blue to colourless and finally spectrophotometrically at 600 nm at the end of the culturing [37].

Percentage biodegradation was calculated by:

$$\%degradation=\left(1-\frac{A_{600nm\left(Treatment\right)}}{A_{600nm\left(Control\right)}}\right)\times 100$$

#### 2.4.3 16S rRNA and catechol 2,3-dioxygenase (*C23O*) gene profiling

DNA was extracted from pure bacterial isolates using the Quick g-DNA Extraction Kit™ (Zymo Research Corporation, CA, USA) according to the manufacturers' instruction and stored at −20 °C prior to further analysis. PCR amplification of the whole variable region of bacterial 16S rRNA was done using the forward primer 27F (5′-AGAGTTTGATCCTGGCTCAG-3′) and the reverse primer 1492R (5′-TACGGYTACCTTGTTACGACTT-3′). Each 25 μL reaction volume contained 0.5 μM of each primer, 1X OneTaq® Hot Start Master Mix (New England Biolabs, Ipswich, MA, USA) and 20 ng DNA. PCR was performed under following cycling conditions (95 °C, 5 min; 32 x [95 °C, 1 min; 55 °C, 1 min; 72 °C, 1 min]; 72 °C, 7 min; 4 °C, ∞), and resultant amplicons checked on a 1.5% agarose gel. The resultant PCR products were purified with ZR DNA Clean and Concentrator Kit (Zymo Research Corporation, CA, USA) according to the manufacturer's instructions and sequenced an ABI-3730 DNA Analyzer (Inqaba Biotech, Pretoria, South Africa). All the 16S rRNA sequences were checked and edited with BioEdit software to manually correct the chromatograms obtained from the Sanger sequencing. Prior to constructing phylogenetic tree, sequences and their top BLAST hit in NCBI database were aligned using CLUSTAL-W. Phylogenetic tree was then constructed using the maximum likelihood (ML) algorithm in MEGA7 as described previously [38].

To further characterize the crude oil sludge degrading bacteria, PCR amplification of the extradiol ring-cleavage catechol 2,3-dioxygenase (C23O) was performed using specific primers C23OF (5′-AAG AGG CAT GGG GGC GCA CCG GTT CGA-3′) and C23OR (5′-TCA CCA GCA AAC ACC TCG TTG CGG TTG CC-3′) in a 25 μl reaction mixture [0.5 μM of each primer, 1X OneTaq® Hot Start Master Mix (New England Biolabs, Ipswich, MA, USA) and 20 ng DNA]. The PCR conditions included: 1 cycle at 98 °C for 10s, then 34 cycles at 98 °C for 1s, 55 °C for 1 min and 72 °C for 15s. Then, a final elongation stage at 72 °C for 1 min. The resultant PCR fragment size (912 bp) spanning the open reading frame (ORF) of the *C23O* gene [39], was visualized by agarose gel electrophoresis. *E coli* DHα lacking the ability to utilise PAHs in crude oil sludge was used as a negative control.

### 2.5 Crude oil sludge and PAHs degradation by bacterial consortia

A consortia consisting of 34 bacterial isolates exhibiting crude oil waste degrading ability was prepared by initially culturing each colony on nutrient broth overnight at 37 °C. After 24 h, 200 μL of each enriched were added simultaneously to 100 mL MSM broth supplemented with 5% crude oil waste sludge and the media incubated at 28 °C for 24 h at 120 rpm for 30 days. The experiment was performed in duplicate, and uninoculated flasks considered as controls. After 30 days, 5 mL of the bacterial consortia was resubcultured on fresh 100 mL MSM containing 5% crude oil waste sludge for 24 h at 28 °C while shaking at 120 rpm for 30 days. Similar experiments were performed using pyrene and anthracene as the sole carbon source. Using GC-MSD, the residual concentrations of the crude oil sludge and its constituent PAHs was determined. The total DNA of the enriched bacterial consortium at the end of the experiments was also extracted and subjected to Illumina Miseq sequencing to establish the bacterial community diversity and composition as described in subsection 2.3.1 and 2.3.2.

### 2.6 Data availability

All the raw datasets from Illumina sequencing have been deposited at the NCBI database (https://www.ncbi.nlm.nih.gov/) sequence archive (SRA) as BioProject ID PRJNA794053. The 16S rDNA sequences were also deposited at the NCBI GenBank database under accession numbers MK854826 - MK854993. The data analysis results obtained during this study are included in the manuscript.

## 3 Results

### 3.1 Changes in physicochemical conditions during co-composting

**Figure 1:**
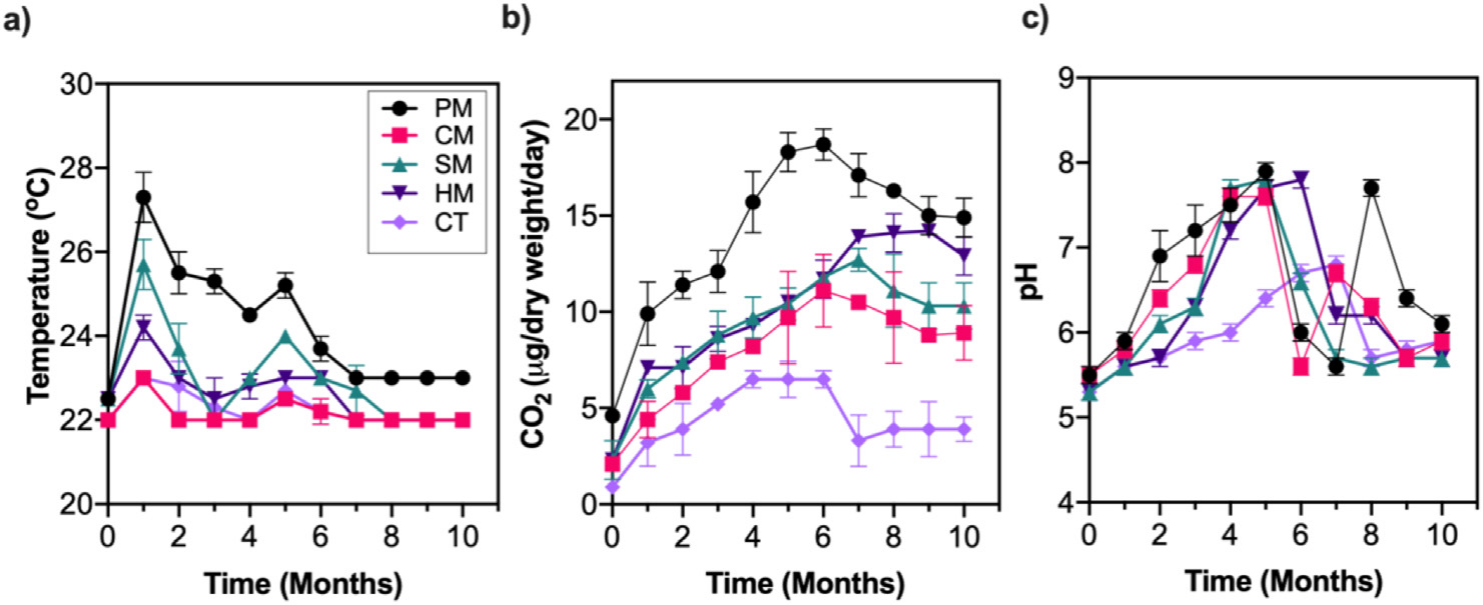
The changes in physicochemical properties during 300 days co-composting treatments of crude oil waste sludge with different manures. (a) Temperature, (b) respiration rate and (c) pH. Soil-crude oil sludge mixture (SSM) + wood chips amended with poultry manure (PM); horse manure (HM); cow manure (CM); pig manure (SM), and no manure amendment (CT).

Changes in temperature, CO_2_ evolution (respiration rate) and pH are presented in Figure 1. Overall, higher temperature and respiration rates were recorded in all the composts piles amended with manure than the control treatment (CT). However, PM showed a higher temperature than other manure-amended treatments, recording peak mean temperature of (27.3 ± 0.6 °C) after 1 month, before fluctuating to 25.2 ± 0.3 and 23.0 ± 0.1 °C after 5 and 10 months of co-composting, respectively (Figure 1a). In contrast, CM and CT recorded the least temperature changes during composting. Similarly, PM exhibited significantly higher CO_2_ evolution (∼18 μg/dry weight/day) after 5 months of composting, with other manure compost piles (HM, CM and SM) recording moderate CO_2_ evolution (∼10 μg/dry weight/day) during the same period (Figure 1b). The control treatment (CT), exhibited relatively lower CO_2_ evolution values during the whole co-composting period. These results showed that manure treatments accelerated the rate of temperature and respiration rate increase.

During the composting, the pH in PM, CM, SM, HM and CT microcosm increased from 5.9-7.9, 5.8–7.6, 5.6–7.8, 5.6–7.7 and 5.6–6.8, respectively (Figure 1c). There was an overall trend of slight increase in pH for all treatments during the first five months, before fluctuating to pre-composting values after 8 months. The only exception was PM treatment that exhibited a sharp increase in pH to 7.9 in the eighth month, before finally fluctuating to pH 6.1 at the end of composting (10 months).

### 3.2 Crude oil waste sludge PAHs reduction during composting

**Figure 2:**
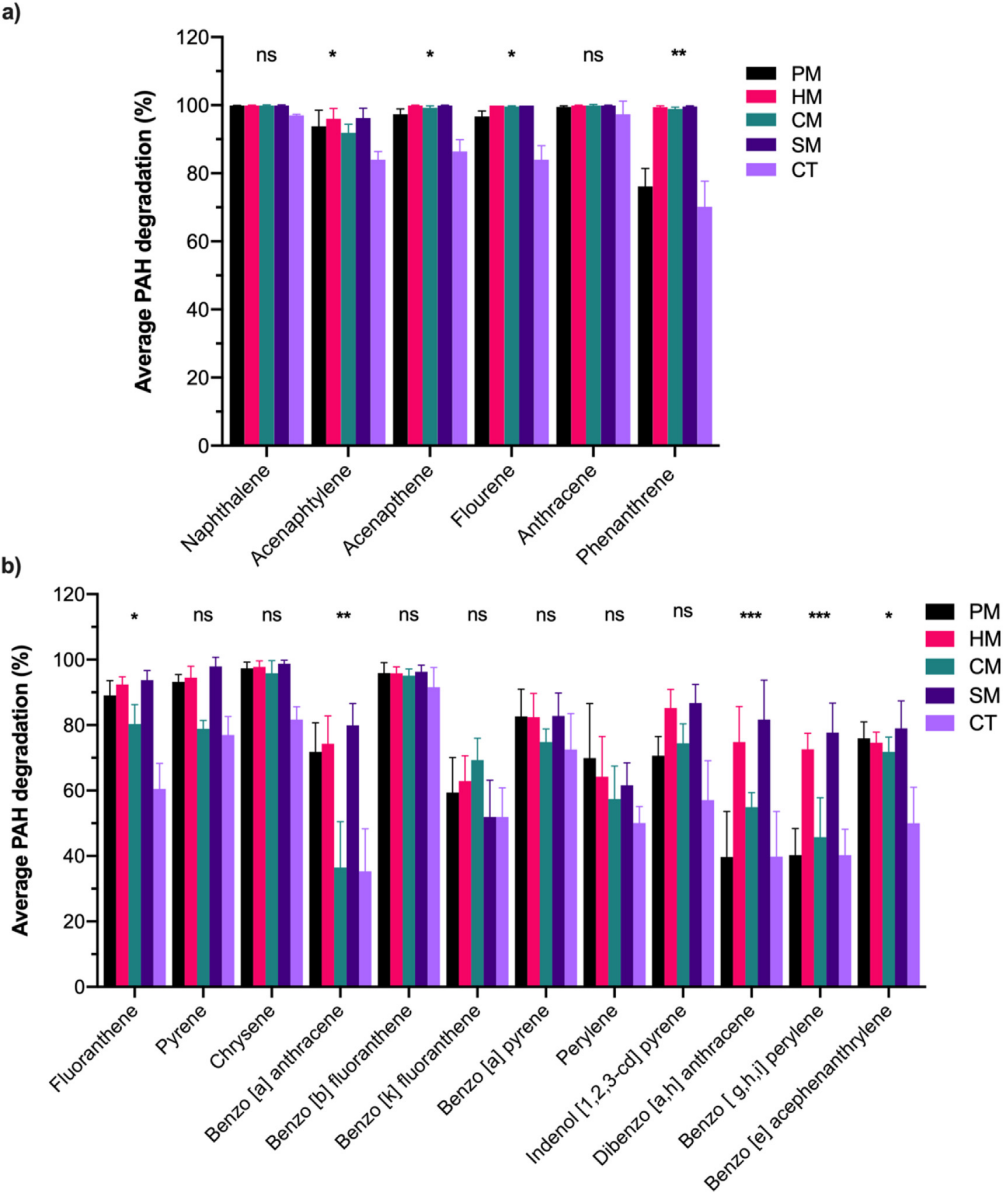
The average reduction of selected crude oil sludge PAHs in poultry (PM), horse (HM), cow (CM), pig (SM), and Control (CT) compost mixtures after 300 days. Degradation of low molecular weight (LMW-PAHs; 2–3 rings) (a) and high molecular weight (HMW-PAHs; 4–6 rings) (b). Error bars represent the standard error of the mean of triplicate microcosms.

GC/MS analysis identified 18 PAHs in crude oil waste sludge ranging from low- (LMW) to high molecular weight (HMW) compounds. The average reduction levels of each PAH in the samples after composting with different manure amendment is presented in Figure 2.

Overall, 36.5–99.9% PAH reduction levels were achieved under the composting conditions with reduction efficiency depending on the compost treatment and the molecular weight of the PAHs. Compost treatments, irrespective of the manure type, resulted in comparatively higher reduction of total PAHs (ΣPAHs), LMW- and HMW-PAHs than in control samples. Manure microcosms (PM, HM, SM and CM) had >300 mg/kg soil ΣPAHs reduction compared to control (CT) 175.8 mg/kg soil. HMW-PAHs such as perylene, dibenzo[a,h]anthacene, benzo[ghi]perylene and benzo[e]acephenathrylene exhibited lower reduction (39.7–81.7%) (Figure 2b), whereas reduction levels up to 99.9% was achieved for LMW-PAHs such as naphthalene, acenaphthene, anthracene, fluorene and phenanthrene (Figure 2a).

### 3.3 Bacterial community diversity during co-composting of crude oil waste sludge

**Figure 3:**
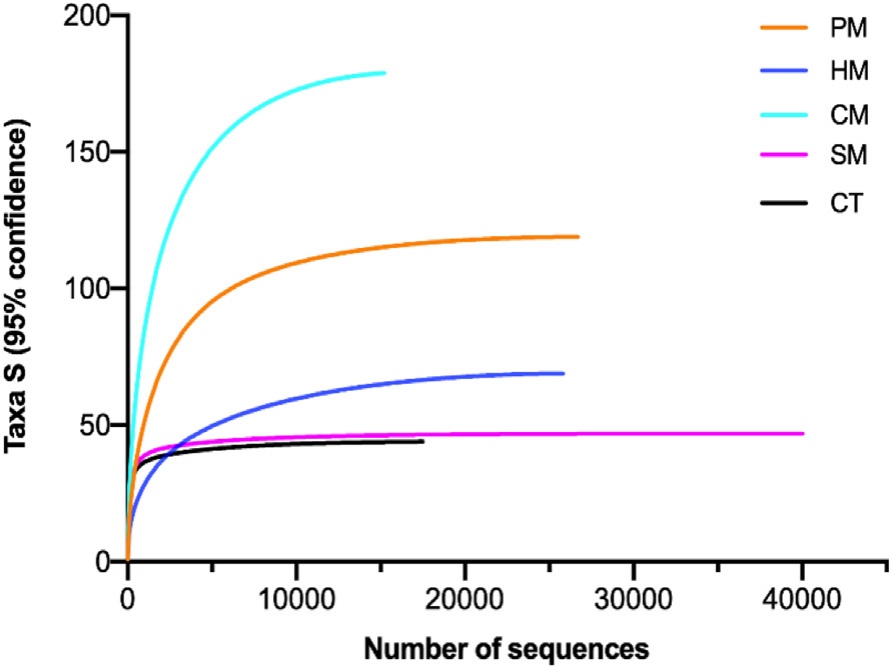
Rarefaction curves indicating the observed number of OTUs within the 16S rRNA gene sequences of crude oil sludge samples composted using different manures. OTUs are shown at the 3% genetic distance levels.

Summary of the sequencing outputs and diversity indices for bacterial communities in co-composting experiments is presented in Table 2. Overall, a total of 125,972 high quality reads (ranging from 15,377 to 40,129) with an average read length of 527 bp were obtained based on 16S rDNA amplicon sequencing analysis. Good's coverage across the samples was >98.5%. This indicated that the sampling depth was sufficient to estimate the microbial diversity enclosing all major bacterial groups involved in co-composting of crude oil waste sludge. This was further supported by the rarefaction curves (Figure 3) that asymptotically approached a plateau, suggesting that the sampling depth accurately reflected the bacterial communities. Comparatively, higher species richness estimates (OTUs and *Chao-1*) were observed for PM and CM samples than HM and SM treatments (Table 2). Additionally, *Chao-1* index revealed lower species richness in CT samples, whereas higher bacterial diversity (Shannon_H) was observed for HM followed by PM, with SM and CM exhibiting comparable values to CT samples.

**Table 2:** Summary of sequencing outputs and diversity indices for bacterial communities in composting experiments of crude oil sludge using different manures.^a^

Indices†	PM	HM	CM	SM	CT
OTU	119	69	179	47	44
Target reads	26,884	25,972	15,377	40,129	17,610
Dominance_D	0.29 (0.282–0.291)	0.51 (0.503–0.518)	0.13 (0.131–0.138)	0.12 (0.119–0.122)	0.11 (0.113–0.119)
Simpson_1-D	0.71 (0.709–0.718)	0.49 (0.482–0.497)	0.87 (0.862–0.0.869)	0.88 (0.878–0.881)	0.88 (0.881–0.887)
Shannon_H	1.91 (1.885–1.926)	1.27 (1.253–1.292)	2.83 (2.809–2.865)	2.65 (2.639–2.664)	2.76 (2.737–2.774)
Evenness_eˆH/S	0.06 (0.055–0.058)	0.05 (0.051–0.053)	0.10 (0.098–0.098)	0.30 (0.298–0.305)	0.36 (0.351–0.364)
Chao-1	119 (119.2–128.2)	69 (69.3–80.0)	180 (180.8–196.3)	47 (47–48)	44(44–47)
Good's coverage (%)	99.7	98.5	99.2	98.6	99.0

a PM – poultry manure; HM – horse manure; CM – cow manure; SM – swine/pig manure; and CT – control (CT = enrichment sample having no manure supplementation).

† Chao-1, community richness-higher number represents more richness; Shannon_H, community diversity-higher number represents more diversity; coverage, sampling depth; OTUs, Operational taxonomic units.

### 3.4 Distribution of taxa and phylotypes in co-composted samples

**Figure 4:**
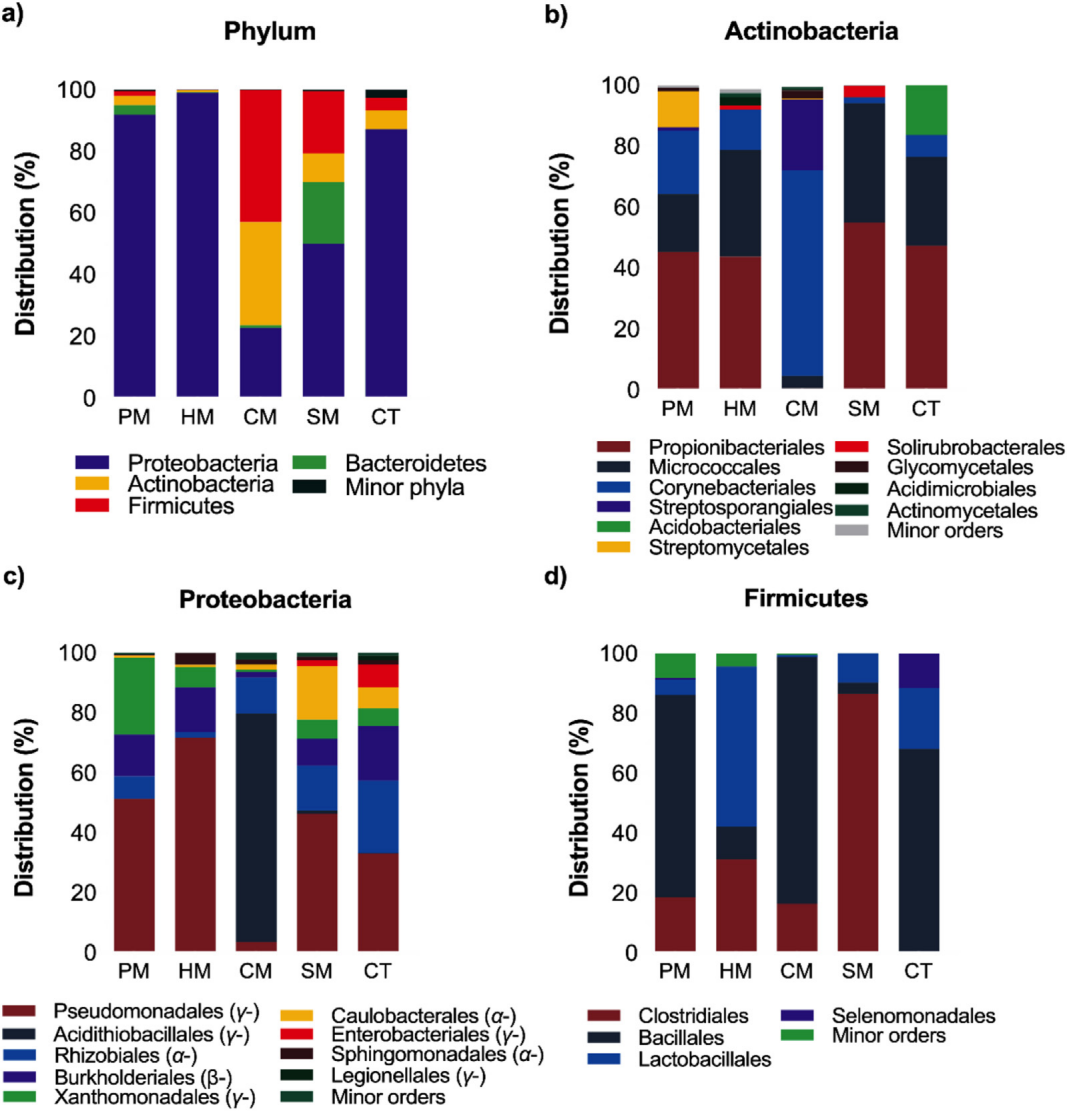
Comparative taxonomic distribution of bacterial communities in crude oil sludge composting treatment with different manures. Diversity at phylum level (a) and the dominant orders in the three most abundant bacterial phyla is illustrated (b-d). The classification of the dominant bacterial orders belonging to phylum *Proteobacteria* into α-, β- and γ-class lineage is shown in brackets (c). Soil-crude oil sludge mixture (SSM) + wood chips amended with poultry manure (PM); horse manure (HM); cow manure (CM); pig manure (SM), and no manure amendment (CT).

In total, 17 phyla, 42 classes, 83 orders, 162 families and 359 genera were detected in the study. OTUs assigned to the phylum *Proteobacteria*, *Firmicutes*, *Actinobacteria* and *Bacteroidetes* were dominant taxa, accounting for >99% of sequences across the co-composting treatment (Figure 4a). However, subtle variation in the relative distribution of bacterial phyla in each co-composting treatment was discernible. Comparatively, members of *Proteobacteria* were highly enriched in HM (99%), PM (90%), CT (85%), SM (50%). CM was dominated by *Actinobacteria* (35%) and *Firmicutes* (45%) with members of phyla *Proteobacteria* accounting for 20% of the detected sequences (Figure 4a). In contrast, *Bacteroidetes* were only detected in SM (20%) and PM (5%). Other minor phyla detected included *Verrucomicrobia*, TM6_(Dependetiae), *Acidobacteria*, *Saccharibacteria*, *Tenericutes*, *Spirochaetae*, *Cyanobacteria*, BRC1 and *Chloroflexi*. At class level, γ-proteobacteria, α-proteobacteria, Actinobacteria, *β-proteobacteria, Clostridia, Bacilli, Bacteroidiia* and *Sphingobacteriia* were the dominant groups in all samples.

The relative distribution of the bacterial orders belonging to the top three phyla is illustrated in Figure 4b, c and d. Generally, the relative abundance of each bacterial taxonomic group varied among the co-composting treatments. Order *Propionibacteriales* and *Micrococcales* was highly enriched in most co-composting treatments (SM, CT, PM and HM) accounting for >45 and >19% relative abundance, respectively, of *Actinobacteria* detected. Interestingly, CM was enriched with *Corynebacteriales* (67.4%) and *Streptosporangiales* (23.1%), whereas members of the orders *Propionibacteriales* and *Micrococcales* were detected at 0.7 and 4.0% relative abundance, respectively (Figure 4a). Another notable observation was detection of members of order *Streptomycetales* (11.7% relative abundance) in PM treatment only. On the other hand, members of the order *Acidithiobacillales* were highly enriched in CM (80%). In contrast, order *Pseudomonadales* was relatively more abundant in most co-composting treatments including control samples, but very low (3% relative abundance) in CM. Other important orders detected included *Rhizobiales, Burkholderiales, Xanthomonadales, Caulobacterales, and Enterobacteriales* (only detected in SM and CT) (Figure 4c). Figure 4d also shows that members of order *Clostridiales* accounted for 90% of *Firmicutes* taxa in SM, whereas *Bacillales* was dominant in all samples with exception of SM. However, only members of *Bacillales*, *Lactobacillales* and *Selenomonadales* (10% only detected in CT) were detected in CT sample.

**Figure 5:**
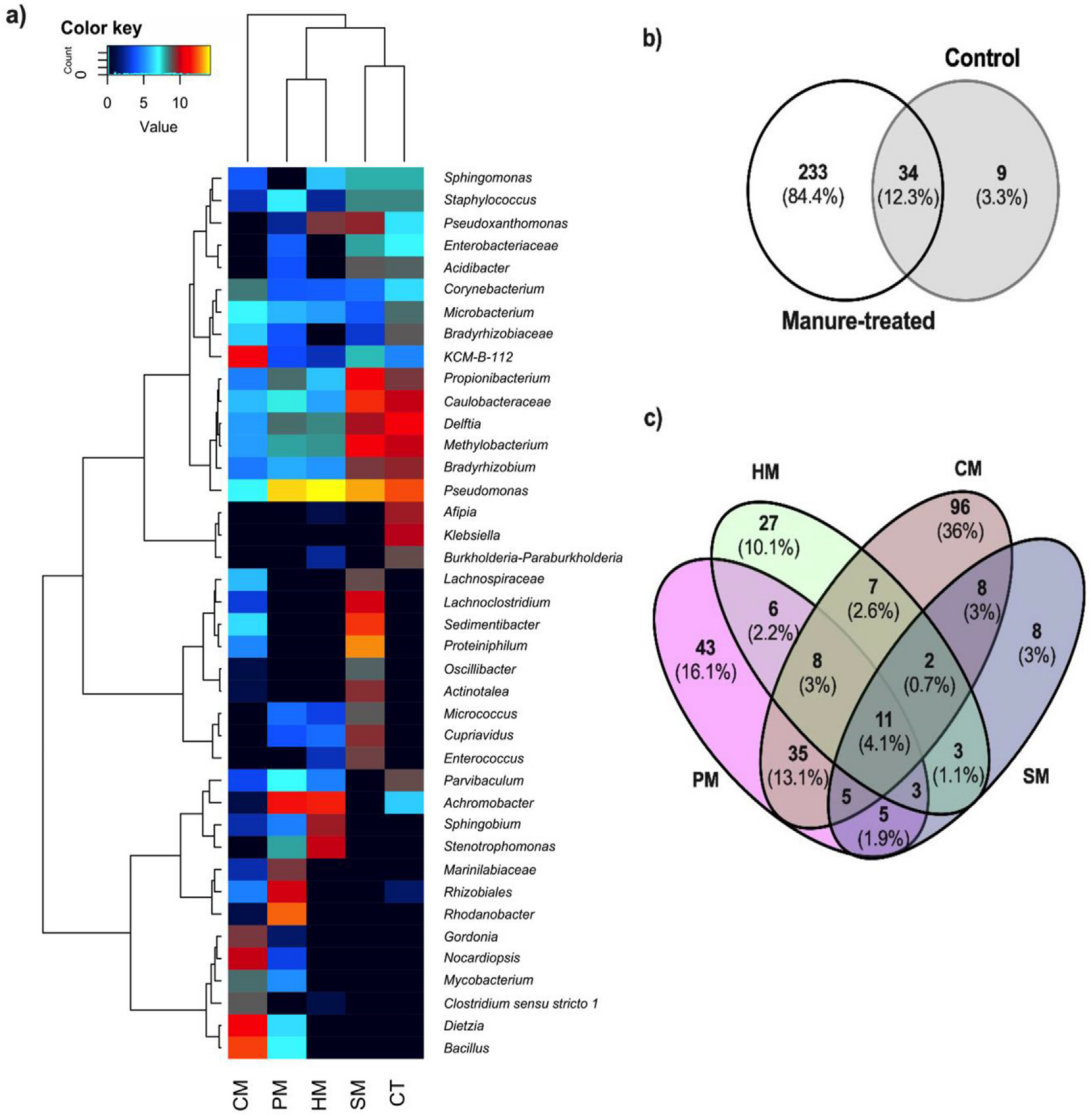
Relative taxonomic distribution of genera and common microbiome diversity in the composting treatment of crude oil sludge. a) Heatmap of the normalized abundance at the genus level for bacteria in the six microcosm metagenomic sequences. Colour code based on higher (yellow) or low (black) relative abundance in metagenomes (see scale on the top left). Venn diagrams representing unique and shared bacterial OTUs between manure-amended and control treatments (b) and within the four manure-treated samples (c).

At genus level, *Pseudomonas, Rhodanobacter*, *Proteiniphilum*, *Enterobacter*, *Achromabacter, Delftia*, *Bacillus*, and *Methylobacterium* were dominant groups (Figure 5a). Overall, the bacterial community profiles of the main genera were clustered into three groups, whereby the bacterial community structures in PM and HM, SM and CT differed remarkably from CM. Comparatively, genera *Pseudomonas* (49.0%), *Rhodanobacter* (22.6%), *Achromobacter* (10.7%), unclassified *Rhizobiales* (4.6%), *Marinilabiaceae_ge* (2.2%) and *Propionbacterium* (1.4%) were highly enriched in PM, whereas *Pseudomonas* (70.2%), *Achromobacter* (12.0%), *Stenotrophomonas* (4.5%), *Sphingobium* (3.2%), *Pseudoxanthomonas* (2.3%), *Delftia* (1.2%) *Massilia* (1.2%) and *Methylobacterium* (1.1%) were the main genera in HM. By contrast, genera *Bacillus* (17.4%), *KCM-B-112_ge* (11.3%), *Nocardiopsis* (7.6%), *Gordonia* (4.0%), *Clostridium sensu stricto_1* (3.0%), *Mycobacterium* (2.4%), *Corynebacterium* (2.2%) and *Rhodococcus* (1.8%) were enriched in CM treatment.

### 3.5 Venn diagram analysis of the variations in taxonomic groups

According to Venn diagrams consistent overlap patterns of genus clusters among different manure-treated co-composting and control treatment of crude oil waste sludge were obtained (Figure 5b). The shared OTUs between manure-treated and control samples were 34 (12.3%), mainly assigned to genus *Pseudomonas*, *Delftia*, *Methylobacterium*, *Dietzia*, *Bacillus*, *Propionibacterium*, *Bradyrhizobium*, *Streptomyces*, *Achromobacter, Microbacterium* and *Sphingomon*as. However, manure-treated had the highest unique OTUs with 233 (84.4%), while CT had 9 (3.3%). The major unique genera in manure-treated samples included *Proteiniphilum*, unclassified *Micrococcales*, unclassified *Lachnospiraceae*, *Sphingobium* and *Stenotrophomonas*, whereas *Afpia*, *Klebsiella* and *Burkholderia-Paraburkholderia* were dominant unique genera in CT. The Venn diagram of the shared and unique microbiome in manure-treated samples is presented in Figure 5c. Manure-treated compost treatments shared 11 OTUs (4.1%), with HM, CM, SM and PM having 27, 96, 8 and 43 unique OTUs detected, respectively.

### 3.6 Isolation of culturable bacteria from co-composting samples

**Figure 6:**
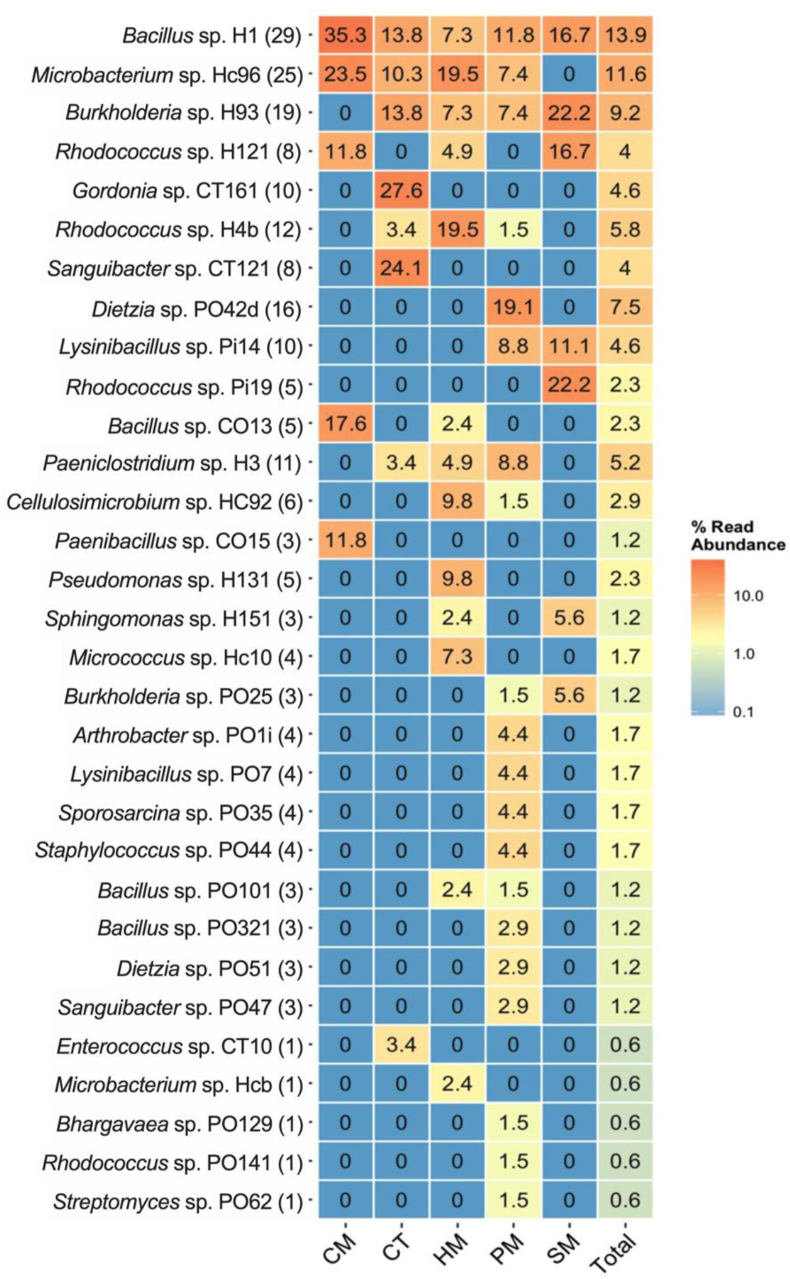
The relative abundance of 31 bacterial phylotypes (based on OTUs) isolated from cow (CM), horse (HM), poultry (PM) and pig (SM) manure amended samples after 10 months composting treatment of crude oil sludge. The putative identity based on 16S rDNA sequence of representative isolate and total number of isolates recovered for each phylotype is provided in the brackets.

To augment metagenomic-based studies, axenic culture studies were performed. The average number of culturable bacteria for manure-treated compost was 4.1 × 10^3^ CFU/g, with PM and CT yielding the highest (389,089 CFU/g) and lowest counts (1,040 CFU/g), respectively. A total of 211 bacterial colonies were picked from MSA plates supplemented with crude oil waste sludge as a sole C-source, and were identified based on partial 16S rDNA sequence to putatively belong to 31 phylotypes based on OTUs. A heatmap showing the relative abundance of the bacterial phylotypes recovered from the different manure and control samples after 10 months composting of crude oil waste sludge is presented in Figure 6.

Overall, phylotypes belonging to genera *Bacillus*, *Lysinibacillus*, *Microbacterium*, *Burkholderia*, *Dietzia*, *Rhodococcus*, *Pseudomonas and Paeniclostridium* were recovered in high frequency (>4% relative abundance) across all samples. However, only five isolates belonging to 4 genera: *Bacillus; Microbacterium; Rhodococcus; and Paenibacillus* were recovered in CM. In contrast, members of genera *Burkholderia*, *Bacillus, Gordonia*, *Enterococcus*, *Geobacillus*, *Sanguibacter* and *Bhargavaea* were isolated from control (CT) samples. Overall, higher species diversity and number of isolates (n = 119) were recovered in PM samples, with phylotypes belonging to genera *Dietzia*, *Burkholderia, Arthrobacter*, *Lysinibacillus, Sporosarcina*, *Staphylococcus*, *Sanguibacter*, *Bhargavaea Rhodococcus* and *Streptomyces* were unique to PM samples only (Figure 6). In contrast, HM, SM and CT exhibited moderate species diversity among the isolates, with CM reporting the least.

### 3.7 PAH biodegradation and cell viability of bacterial isolates within a microcosm setup

In order to perform PAH-degrading tests, 93 putative hydrocarbonoclastic bacterial isolates were chosen based on colour and colony morphology differences. A microcosm-based strategy was implemented to measure the ability of the axenic cultures to grow, utilise and degrade target PAHs, using crude oil waste sludge as the sole carbon or energy source. The activity was screened colorimetrically using redox indicator 2,6-dichlorophenol indophenol (2,6-DCPIP). The ability of each axenic culture to degrade the crude oil waste sludge was considered to be proportional to the decolourization of DCPIP incorporated into the growth media.

**Figure 7:**
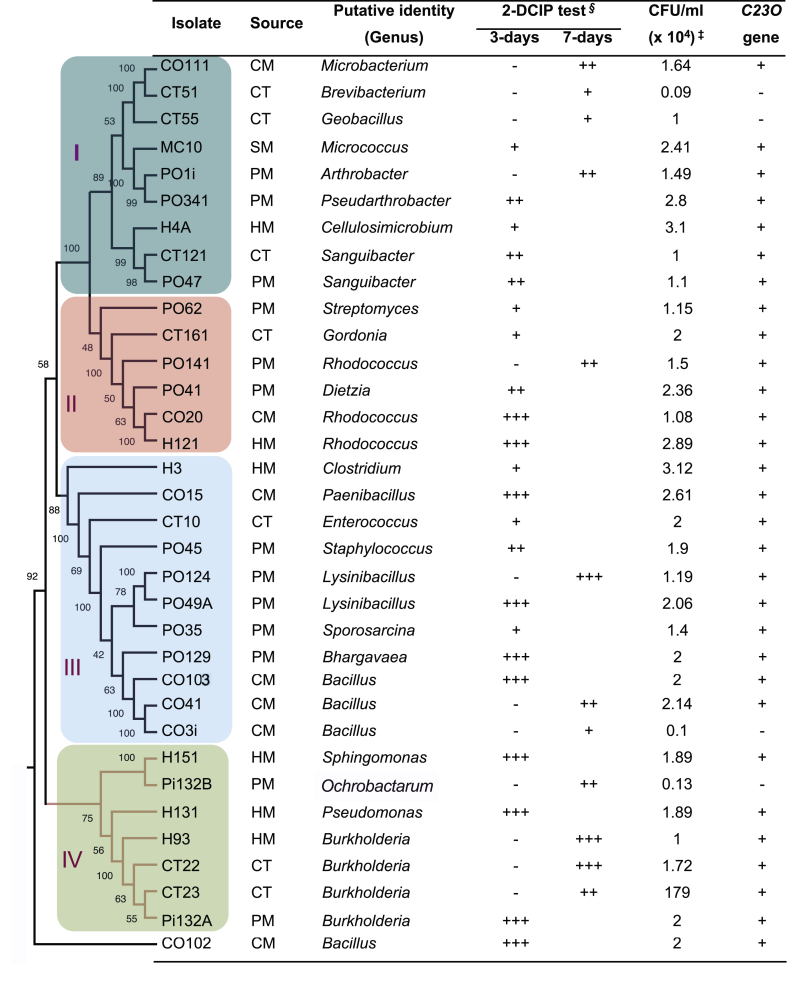
The putative PAH-degrading bacteria isolated from compositing treatment of crude oil sludge using different manures. The bacterial isolates were identified by 16S rDNA sequencing (average length 1450 bp) and used for constructing the cladogram. The percentage of replicate trees in which the associated taxa clustered together in the bootstrap test (1000 replicates) are shown next to the branches. The four major phylogenetic clades (I, II, III and IV) of PAH-degrading bacteria is illustrated using different colors. The putative identity at genera level, 2,6-DCPIP tests, plate counts and in the presence of catechol 2,3-dioxygenase is provided. Rating based on the loss of 2-DCPIP indicator blue color due transfer of electrons from the degradation of PAHs by bacteria: -, no change; +, minimal; ++, moderate; and +++, complete color change to colorless. ‡ Plate counts after culturing in MSM-crude oil sludge for 30 days. The assays were performed in biological triplicates.

Axenic cultures of thirty-three isolates were qualitative positive for crude oil waste sludge degradation, with 6, 7, 6, 1 and 13 recovered from CM, CT, HM, SM and PM microcosms, respectively (Figure 7). These isolates were also recovered after 30 days treatment using crude oil waste sludge as sole carbon source, yielding 0.1-3.12 × 10^4^ CFU/g culturable bacteria. Furthermore, 29 out of 33 isolates were positive for catechol 2,3-dioxygenase gene (C23O). Cultures found to be efficient degraders of the crude oil sludge could be phylogenetically grouped into 4 clades (Figure 7). Clade I consisted members of order *Micrococcales* (genus *Microbacterium*, *Brevibacterium*, *Geobacillus*, *Micrococcus*, *Arthrobacter*, *Sanguibacter* and *Cellulimicrobacterium*) belonging to the phylum *Actinobacteria*. These isolates were recovered from all treatments, including the control (CT) samples, with exception of swine/pig manure (SM). In clade II, six isolates belonging to orders *Streptomycetales* (genus *Streptomyces*) and *Corynebacteriales* (genus *Dietzia*, *Rhodococcus*, and *Gordonia*) within the phylum *Actinobacteria*. In contrast, Clade III were mostly members of th phylum Firmicutes. The bacterial genus *Bacillus* and *Lysinibacillus* were the most recovered group in this study. Others included *Clostridium, Enterococcus, Staphylococcus, Sporosarcina* and *Bhargavaea*. In clade IV, included isolates members of class *Betaproteobacteria* (genus *Burkholderia*) and *Alphaproteobacteria* (genus *Sphingomonas* and *Ochrobactarum*). Interestingly, one novel PAH-degrading bacterial isolate that exhibited the highest similarity of ∼76.7% to members of genus *Bacillus* was also recovered from cow manure (CM) sample. These bacterial isolates were the most efficient degraders of the crude oil sludge. The evolutionary relationship of these isolates and their GenBank relatives are displayed in Table 3.

**Table 3:** Molecular identification of the putative PAH-degrading bacterial isolates. All sequences were compared with reference 16S rRNA gene sequences available in the GenBank/EMBL/DDBJ databases using BLAST. The accession number to the NCBI, the closest type strain and the corresponding sequence is listed.

Isolate	Accession NCBI	Closest Type Strain	Reference Sequence	Similarity (%)	Source of type strain
CO15	MK854828.1	*Paenibacillus* lautus strain NBRC 13380^(T)^	NR_112724.1	99.8	Soil
C0102	MK854981.1	*Bacillus pumilus* isolate El-24-8	AJ494726.1	76.8	Marine sediments
CT51	MK854979.1	*Microbacterium hominis* strain DSM 12509	NR_042480.1	97.3	Lung aspirate
CT55	MK854980.1	*Microbacterium hominis* strain DSM 12509	NR_042480.1	97.3	Lung aspirate
MC10	MK854858.1	*Micrococcus luteus* strain NCCP 16831	CP043842.1	99.7	human
H121	MK854848.1	*Rhodococcus soli* strain DSD51W	NR_134799.2	98.8	Park soils
CT121	MK854971.1	*Sanguibacter soli* strain DCY22	NR_044276.1	99.5	Ginseng field
CT61	MK854986.1	*Gordonia amicalis* strain IEGM 1273	NR_028735.1	99.5	Oil contaminated soil
H151	MK854850.1	*Sphingopyxis bauzanensis* strain BZ30	NR_117213.1	99.3	Oil contaminated soil
H3	MK854922.1	*Paeniclostridium sordelli* strain JCM 3814^(T)^	NR_113140.1	99.5	Marine sediment
H4b	MK854850.1	*Rhodococcus degradans* strain CCM 4446	NR_043535.1	100	Contaminated soil
H93	MK854855.1	*Burkholderia lata* strain 383	NR_102890.1	99.9	Forest soils
Hc10	MK854858.1	*Micrococcus aloeverae* strain DSM 27472^(T)^	NR_075062.2	99.8	Aloe vera tissues
H4a	MK854951.1	*Cellulosimicrobium funkei* strain W6122	NR_042937.1	99.3	Blood
PO101	MK854904.1	*Bacillus kochii* strain WCC 4582^(T)^	NR_117050.1	98.5	Foods, pharmaceutical manufacturing site
PO341	MK854924.1	*Pseudoarthrobacter oxydans* strain DSM 20119	NR_026236.1	99.8	Air
PO41	MK854926.1	*Dietzia maris* strain DSM 43672	NR_037025.1	99.0	Open soil
CO20	MK854831.1	*Rhodococcus hoagiii* strain ATCC 6939	NR_116691.1	99.9	Horse
PO129	MK854949.1	*Bhargavaea beijingensis* strain ge10	NR_117988.1	99.1	Ginseng root
CT10	MK854970.1	*Enterococcus mundtii* strain DSM 4838	CP018061.1	99.4	Soil
PO45	MK854932.1	*Staphylococcus succinus* strain 14BME20	CP018199.1	100	Fermented soybean food
PO124	MK854908.1	*Lysinibacillus fusiformis* strain DSM2898	NR_042072.1	100	Open soil
PO1i	MK854914.1	*Arthrobacter tecti* strain LMG 22282	NR_042251.1	99.2	Deteriorated mural paintings
PO35	MK854925.1	*Sporosarcina luteola strain* NBRC 105378^(T)^	NR_114283.1	99.7	Sea water
PO42d	MK854965.1	*Dietzia maris strain* DSM 436782	NR_118596.1	100	Open soil
PO49a	MK854936.1	*Lysinibacillus pakistanensis* strain NCCP-54	NR_113166.1	99.5	Soybean rhizosphere
PO35	MK854925.1	*Sporosarcina luteola* strain NBRC 105378	NR_114283.1	99.7	Soy sauce
*PO44*	MK854931.1	*Staphylococcus epidermis* strain 10091	NR_0369904	99.3	Skin
CO41	MK854834.1	Bacillis *subtilis* strain IAM 12118	NR_112116.2	99.9	Open soil
PO47	MK855493.1	*Sanguibacter marinus* strain 1-19	NR_042311.1	96.6	Coastal sediments
PO62	MK854943.1	*Streptomyces pseudogriseolus* strain NRRL B-3288	NR_043835.1	99.2	Soil
PO7	MK854944.1	*Lysinibacillus pakistanensis* strain NCCP-54	NR_113166.1	99.5	Soyabean rhizosphere
CO3i	MK854833.1	*Bacillus zhangzhouensis* strain MCCC 1A08372	NR_148786.1	99.7	Aquaculture water
Pi131b	MK854890.1	*Ochrobactrum pecoris* strain 08RB2639	NR_117053.1	100	Sheep
H131	MK854849.1	*Pseudomonas chloritidismutans* strain AW-1	NR_115115.1	98.4	Anaerobic chlorate-reducing bioreactor
CT22	MK854978.1	*Burkholderia metallica* strain R-16017	NR_042636.1	99.7	Plants
Pi132a	MK854889.1	*Burkholderia lata* strain 383	NR_104978.1	99.7	Forest soil

### 3.8 Microbial bioremediation of crude oil sludge PAHs using the enriched bacterial consortium

**Figure 8:**
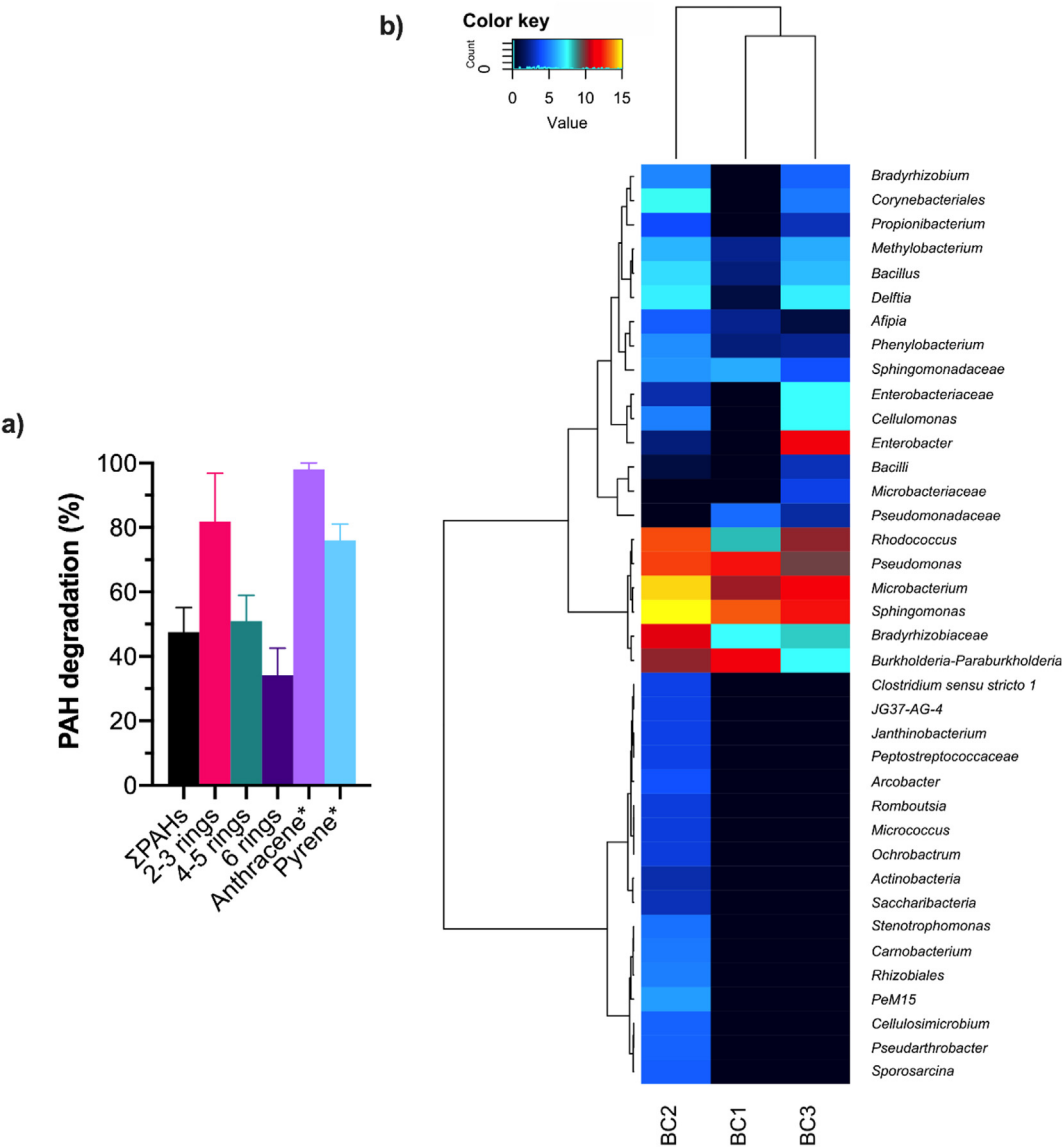
Bacterial consortium utility for bioremediation of crude oil sludge waste PAHs. a) PAH content by molecular weight on day 30. Error bars represent the standard deviation of the mean of triplicate microcosms. b) Heatmap the normalized abundance at the genus level for bacteria in the three microcosms metagenomic sequences (BCI, BC2 and BC3 included culture media supplemented with crude oil sludge, pyrene and anthracene, respectively, as sole carbon source) on day 30 based on the weighted UniFrac distance.

In this study, an enriched consortia of the 33 efficient PAH-degraders were added to crude oil sludge (BC1)-, pyrene (BC2)- and anthracene-contaminated (BC3) media to test their bioaugmentation capacity. The PAHs were degraded gradually in media; total PAH (ΣPAHs), pyrene and anthracene contents dropped from 359.7 to 106.7, 14.0 to 3.36 and 42.0 to 0.084 mg/kg, respectively, after 30 days of treatment (Figure 8a). The removal rates were 39.6–54.8, 77.8–98.3, 43.2–58.6, and 26.4–44.7% for ΣPAHs, two-to three, three-to five and six-ring PAHs were observed in BC1 treatment samples after 30 days treatment. In BC2 and BC3 treatments, higher reduction rates of 72.5–81.6 and 87.8–98.6% were observed for pyrene and anthracene, respectively.

To further gain insight on the viability and antagonistic effects of bacterial isolates during PAHs degradation, a cultivation-independent detection method using 16S rDNA amplicon sequencing was carried out for three microcosms (BCI, BC2 and BC3) at day 30. Heatmap of the normalized abundance at the genus level for bacteria in the three microcosms on day 30 is illustrated in Figure 8b. The result revealed that OTUs assigned to genus *Sphingomonas, Pseudomonas, Microbacterium, Rhodococcocus, Burkholderia-Paraburkholderia*, unclassified *Bradyrhizobiaceae*, *Bacillus, Delftia, Cellulomonas, Enterobacter* and *Aflpia* were relatively viable and dominant across all samples. However, subtle variation in the enrichment of the bacterial groups was observed. Hierarchical clustering based on weighted UniFrac distance showed a clear separation of pyrene treated samples (BC2) from other two treatments. Overall, BC1 had higher species richness (*Chao-1* = 89.1) than other two treatments (*Chao-1* = 17 and 28 for BC1 and BC3, respectively). BC2 also had a higher species diversity and relative abundance of top 40 OTUs recovered at day 30. The most abundant genus observed in BC2 included *Microbacterium, Sphingomonas*, *Rhodococcus*, *Burkholderia-Paraburkholderia* and unclassified *Bradyrhizobiaceae*. Interestingly, OTUs assigned to genera such as *Sporosarcina*, *Pseudoarthrobacter*, *Cellulomicrobium* and *Ochrobactarum* associated with bacterial consortium were only observed in BC2. In contrast, only BC3 exhibited enriched abundance of genus *Enterobacter*.

## 4 Discussion

Bioremediation has been established to be a reliable cost-effective technology for oil spill and crude oil waste remediation [40, 41]. However, its success is generally dependent on the natural microbial community populations, whose remediation capacity is greatly influenced by nutrient availability and other *in situ* and *ex situ* physicochemical and environmental conditions. To improve the natural ability of microorganisms to degrade contaminants in oil associated environments, several successful engineered bioremediation approaches utilizing bioaugmentation (addition of known degraders of the contaminant) and or biostimulation (addition of nutrients in the form of fertilizers) have been reported [40, 41, 42, 43]. In this study, we (i) investigated PAHs degradation potential of autochthonous microbial community via biostimulation and/or bioaugmentation by co-composting crude oil sludge with different manures amendments, (ii) performed survey on the total bacterial community using high-throughput targeted 16S rDNA amplicon sequencing to provide insight on the manure-induced community dynamics in the sludge microbiome during PAH degradation, (iii) carried out the isolation, identification and partial characterization of 93 putative hydrocarbonoclastic bacteria and (iv) narrowed on a simplified 33-strains PAH-degrading bacterial consortium which might be useful in designing bioaugmentation/biostimulation strategy for the treatment of crude oil refinery wastes.

### 4.1 Microcosm-based crude oil sludge PAH degradation under manure treatments

In engineered bioremediation for oil-contaminated soils and refinery wastes, addition of N and P containing fertilisers to alleviate nutrient limitation is key in enhancing microbial activity and concomitant PAHs biodegradation [42, 44]. In this study, four animal manures characterized by variable levels of TN, TP and TOC (Table 1) were used in microcosm-based biostimulation and/or bioaugmentation of crude oil sludge PAHs degradation. Our data demonstrated greater effect of the manure amendments in the stimulation of respiration activities and PAHs degradation rates. This is consistent with findings that addition of organic waste materials such as sewage sludge and soybean meal [45] and farm manures [41] enriched oil contaminated soils and refinery wastes with nutrients, such as P and N, whose limitation is known to slow down biodegradation processes [46]. Among the manure treatments, poultry manure (PM) treatment had a significantly higher CO_2_ evolution (Figure 1a) and higher temperatures (Figure 1b) during composting, recording ∼90% loss in the total PAH (ΣPAHs) after 300 days. The higher temperature observed in the PM amendment could be attributed to its high N and P content and the existence of high diversity and density of microorganisms therein, which may have stimulated microbial growth and PAH degradation activities. These results are consistent with reports of several researchers that have employed the application of poultry droppings for improved bioremediation of oil-polluted environments [41, 47, 48]. Similarly, higher degradation rates leading to total PAH losses >90% and relatively higher CO_2_ evolution was observed for other manure treatments. In contrast, non-manure microcosm (CT) showed a baseline attenuation of 52% for total PAH.

Assessment of the nature of the hydrocarbons within the co-composting microcosms was also done to gain information on possible role of manure amendment in the biostimulation of indigenous microorganisms capacity to efficiently degrade different PAH compounds in the crude oil sludge (Figure 2). GC/MS analysis revealed that the original crude oil sludge was found to primarily consisting of LMW PAHs (2–4 rings; 81.5%) and HMW PAHs (5–6 rings; 18.5%). Following manure amendments, depletion rate between 76.1-99.9% was achieved for LMW-PAHs such as naphthalene, acenaphthene, anthracene, fluorene and phenanthrene (Figure 2a) after 300 days. HMW-PAHs such as perylene, dibenzo [a,h] anthacene, benzo [ghi] perylene and benzo [e] acephenathrylene exhibited lower reduction (39.6–81.7%) (Figure 2b). In contrast, non-manure microcosm recorded similar depletion rate for LMW PAHs (70.2–97.3%), but relatively very low reduction rate (<51.9%) for 5–6 rings PAHs. The recalcitrance of PAHs attributed to number of aromaticity determines the distinctive behaviors during degradation, with 2–4 rings PAHs undergoing faster initial degradation, and followed by five-to six-rings PAHs soon afterward [49]. In this study manure amendment promoted considerable biodegradation of HMW PAHs compared to CT.

### 4.2 Shift in microbial community with manure amendments

Based on Baas-Becking hypothesis that “everything is everywhere, but the environment selects” [50], we envisaged that deeper coverage of the co-composting microcosms would reveal pertinent manure-induced biostimulation and bioaugmentation of *in situ* and *ex situ* bacterial community for the improved crude oil sludge remediation. Metagenomic analysis showed that *Proteobacteria*, *Firmicutes*, *Actinobacteria* and *Bacteroidetes* were the predominant phyla present in all the microcosms at day 300. The shared genera between manure-treated and control microcosm included *Pseudomonas*, *Delftia*, *Methylobacterium*, *Dietzia*, *Bacillus*, *Propionibacterium*, *Bradyrhizobium*, *Streptomyces*, *Achromobacter, Microbacterium* and *Sphingomon*as, some of which has been previously reported in oil-contaminated soils [41, 43, 45], with several members known for their ability to degrade aliphatic and aromatic hydrocarbons [51, 52, 53]. Whereas *Pseudomonas and Sphingomonas* have been previously associated with degradation of LMW PAHs [54, 55], fermentative, CO_2_-assimilating and methanogenic microorganisms (*Bacillus*, *Methylobacterium* and *Achromobacter*) are known to be key players in HMW PAH degradation [55]. In addition, *Bacillus*, *Dietzia* and *Achromobacter* are potent biosurfactant producers that promotes efficient emulsification and eventual biodegradation of HMW PAHs [19, 56]. Bacteria from the genera such as *Delftia*, *Bradyrhizobium*, *Microbacterium* and *Streptomyces* can also decompose various aromatic compounds during denitrification [53, 55, 57]. Persistence of the above-mentioned groups in all microcosm treatments provided clues of their autochthonous nature to either crude oil sludge, soil or wood chips used for the experiments, and the important role they play in the baseline attenuation of crude oil sludge PAHs in absence of manure amendments. Interestingly, the manure-amended treatments were associated with comparatively higher bacterial species richness and diversity estimates than CT (Table 2). Besides the shared bacterial groups, manure-treated microcosms were also characterized by presence of additional potential hydrocarbon-degrading bacterial taxa such as *Proteiniphilum*, unclassified *Micrococcales*, unclassified *Lachnospiraceae*, *Sphingobium* and *Stenotrophomonas* [58]. The observed higher relative abundance of the aforementioned native and introduced bacterial groups due to manure-treatment, therefore, points towards the contribution of manure amendments in the improvement of co-composting microcosm microbial diversity and the associated PAHs degradation.

As shown in Figure 5, subtle variations in the bacterial community diversity were also observed within the manure-treatments. The relationship among the samples using Weighted UniFrac analysis revealed clustering into three distinct groups; the bacterial community structures in PM and HM differing significantly from SM and CM. These results substantiated the fact that the microcosm microbial community structures were impacted differently by the manure amendments. Increase in the members of class *γ-proteobacteria* (*Pseudomonas*, *Rhodanobacter*, *Stenotrophomonas, Pseudoxanthomonas*)*, β-proteobacteria* (*Achromobacter, Massilia,* and *Delftia), α-proteobacteria* (*Sphingobium, Methylobacterium* and unclassified *Rhizobiales*), were prominent in PM and HM, while *Firmicutes* (*Bacillus, KCM-B-112, Nocardiopsis*, *Clostridium sensu stricto 1*, *Mycobacterium*, *Corynebacterium* and *Rhodococcus*) and *Actinobacteria* (*Propionbacterium, Corynebacterium*) were found to predominate CM sample. As *Proteobacteria* are known for their catabolic versatility, genetic plasticity and metabolic diversity leading to broad substrate specificities for several classes of hydrocarbons in environments such as natural oil deposits, asphalt, crude oil, oil sand, oil contaminated water, soil and sludge [59], their enrichment in PM and HM microcosms system may not be unconnected to the manure amendments. Further, the copiotrophic nature of the α, β, and γ classes of *Proteobacteria*, imply that the amendment with manures rich in utilizable C, N and other nutrients could be responsible for the increase in the relative abundance of the phylum *Proteobacteria* in PM and HM systems. In contrast, the genetic plasticity, metabolic versatility and production of extracellular and cellular biosurfactants by members of *Firmicutes* and *Actinobacteria*, have been reported to enhance the uptake and biodegradation of hydrophobic pollutants [60]. This may explain their predominance in CM microcosm.

### 4.3 Identification of PAH-degrading bacteria and potential applications in bioaugmentation

In this study, we also attempted to isolate and identify PAH-degrading bacteria from the co-composting microcosms. A total of 33 putative PAH degraders based on 2,6-DCPIP tests, cell viability and presence of catechol 2,3-dioxygenase gene (*C23O*) (Figure 7) were identified. Consistent with metagenomic results, analysis of the partial 16S rDNA sequences showed that these putative PAH degraders belonged to the genera *Bacillus*, *Lysinibacillus*, *Microbacterium*, *Burkholderia*, *Dietzia*, *Rhodococcus*, *Pseudomonas* and *Paeniclostridium,* taxa that has been previously been isolated from crude oil sludge [61]. The detected GenBank relatives were isolates from sources such as oil contaminated soils, marine sediments, rhizosphere, open field and forest soils (Table 3). The bulk of these bacterial isolates were recovered from PM microcosms, but were heterogeneous across the 4 phylogenetic clades (Figure 7). In contrast, only one isolate assigned to genus *Micrococcus* was recovered SM sample.

Redox indicator 2,6-DCPIP (an electron acceptor) undergoes a colour change from blue (oxidised form) to colourless (reduced form). This color change can be used to determine the capability of microorganisms to utilise and to estimate the PAH biodegradation capacities of axenic bacterial cultures [41, 61]. In this study, all the bacterial isolates tolerated and biodegraded crude oil sludge, positively reacting with the 2,6-DCPIP. However, 9 isolates exhibited very intense and rapid reaction with 2,6-DCPIP, completely changing the color from blue to colourless within 3 days. This included: 3 isolates recovered from CM microcosm assigned to genus *Rhodococcus*, *Paenibacillus* and *Bacillus;* 3 species from HM belonging genus *Rhodococcus, Sphingomonas* and *Pseudomonas; and Lysinibacillus*, *Bhargavaea* and *Burkholderia*. The elevated activities of these isolates in early stages of PAH degradation, indicate that they are fast PAH degraders, mainly associated with degradation of LMW PAHs. In support of these findings, Obi et al. [61] also reported the isolation of *Pseudomonas* and *Bacillus* species from crude oil sludge that had characteristic fast PAH degradation (decolourising the 2,6-DCPIP in the shortest possible time). To complement 2,6-DCPIP test, cell viability test of the isolates after 30 days incubation in MSM media supplemented with crude oil sludge as sole carbon and energy source was undertaken. All isolates yielded between 0.1-3.12 × 10^4^ CFU/g culturable bacteria, indicating their ability to grow and utilize crude oil sludge PAHs. Further, PCR amplification with degenerate primers was also performed to screen for the presence of the cathecol-2,3-dioxygenase (*C23O*) genes in the isolates. The results showed that all the isolates possessed the catabolic *C23O* genes, providing clues on the their potential capability to degrade the crude oil sludge PAHs.

The functional profile of the selected bacterial isolates related to their ability to utilize crude oil PAHs suggests that crude oil sludge-soil-woodchips-manure co-composting microcosms is a reservoir for the recovery of important bacterial species that can be exploited for engineered remediation processes. In this study, an enriched consortia of the identified 33 PAH-degraders was used for laboratory scale bioaugmentation study of crude oil sludge in absence of manure amendments. The treatment resulted in accelerated degradation of total PAHs, anthracene and pyrene within 30 days (Figure 8a), with viability of these strains confirmed by 16S rDNA amplicon high-throughput sequencing. These results demonstrate the potential application of the bacterial consortia as a basic microbial agent for the bioaugmentation of animal manure-treated co-composting bioremediation of crude sludge waste.

## 5 Conclusion

In conclusion, the current study attempted static co-composting technique with different manures to understand the bacterial diversity and its catabolic potential against hydrocarbon degradation using both culture-dependent and culture-independent analysis. Acceleration of respiration rates (CO_2_ evolution) and temperature increase during co-composting indicated that manure treatments enhanced microbial activities which could be linked to overall improvement of PAH degradation. Culture independent analysis revealed that higher bacterial diversity was observed for HM followed by PM, with SM and CM exhibiting comparable values to CT samples. Members of *Proteobacteria* were highly enriched across all samples (HM, 99%; PM, 90%; CT, 85%; and SM, 50%), with exception of CM which was dominated by *Actinobacteria* (35%) and *Firmicutes* (45%) members. Notably, the members of order *Streptomycetales* (11.7%) were only identified in the PM treatment. The major unique genera across all samples were *Pseudomonas*, *Delftia*, *Methylobacterium*, *Dietzia*, *Bacillus*, *Propionibacterium*, *Bradyrhizobium*, *Streptomyces*, *Achromobacter, Microbacterium*, *Sphingomon*as etc., that could be recovered from the microcosm samples by culturing. However, most OTUs were unclassified, which further warrants a comprehensive taxonomic approach to identify their identity and novelty. Many isolated strains were able to grow on crude oil sludge as carbon source, albeit with significant number of strains exhibiting both tolerance and high catabolic activity potential against crude oil sludge PAHs. Therefore, our findings have demonstrated the potential utility of the readily available animal manures as economically feasible co-composting agent for improved bioremediation crude oil sludge waste. Further, this study provides theoretical insights on co-composting with different animal manures as an interesting approach for the exploitation of novel PAH-degrading bacteria, which can further be formulated for engineered bioaugmentation of crude oil pollution remediation.

## Declarations

### Author contribution statement

Onyedikachi Ubani: Conceived and designed the experiments; Performed the experiments; Analyzed and interpreted the data; Wrote the paper.

Harrison Ifeanyichukwu Atagana, Atagana: Conceived and designed the experiments.

Ramganesh Selvarajan: Contributed reagents, materials, analysis tools or data.

Henry JO Ogola: Performed the experiments; Analyzed and interpreted the data; Wrote the paper.

### Funding statement

This work was supported by South Africa 10.13039/501100001321National Research Foundation under the Research and Innovation Support and Advancement (Ph.D Grant, UUID: 89766).

### Data availability statement

Data associated with this study has been deposited at NCBI database (https://www.ncbi.nlm.nih.gov/) sequence archive (SRA) as BioProject ID PRJNA794053 under the accession number MK854826 to MK854993.

### Declaration of interests statement

The authors declare no conflict of interest.

### Additional information

No additional information is available for this paper.
